# A guide to global access to HPV vaccination to all women in low- and middle-income countries; a minireview of innovation and equity

**DOI:** 10.3389/fonc.2024.1380663

**Published:** 2024-10-02

**Authors:** Agnes Ewongwo, Aji Fatou Sahor, Wilfred Ngwa, Chika Nwachukwu

**Affiliations:** ^1^ Department of Radiation Oncology, Stanford University, Palo Alto, CA, United States; ^2^ Department of Obstetrics and Gynecology, University of Texas Health, Houston, TX, United States; ^3^ Department of Radiation Oncology and Molecular Radiation Sciences, Johns Hopkins University, Baltimore, MD, United States; ^4^ Department of Radiation Medicine and Applied Sciences, University of California, San Diego, San Diego, CA, United States

**Keywords:** HPV, cervical cancer, HPV vaccine, low-income country, middle-income country

## Abstract

**Introduction:**

Cervical cancer is caused by the human papillomavirus (HV), and accounts for more than 311,000 preventable deaths annually, with 85% occurring in low-and middle-income countries. Despite being preventable through screening and screening, significant barriers to implementing HPV vaccination persist in developing nations. This review study aims to identify these barriers and propose innovative, evidence-based solutions to improve vaccination rates and reduce cervical cancer mortality.

**Methods:**

A systematic review search was conducted using PubMed, Embase, and Cochrane Database of Systemic Reviews. Keywords related to HPV vaccination barriers and implementation strategies in developing countries were used. Relevant demonstration projects, pilot studies, and evidence-based research articles were reviewed.

**Results:**

Identifiable barriers to a successful vaccine implementation program in a developing country include vaccine costs, societal, cultural resistance, misinformation, logistical challenges in vaccine delivery, and inadequate human resources. Solutions to these barriers include a subsidized vaccine pricing, community sensitization, education and well-trained media professionals to dispel misinformation, and partnerships with both public and private sector for efficient vaccine distribution.

**Discussion:**

These findings highlight critical barriers that impede HPV vaccination efforts in developing countries and offers practical solutions to overcome these challenges. This aggregate of data can help inform future developing countries’ implementation programs to further the World Health Assembly mission to vaccinate 90% of eligible girls globally by 2030.

## Introduction

A woman dies due to cervical cancer every two minutes (1). Cervical cancer causes more than 311,000 preventable deaths per year. 85% of these deaths are in low- and middle-income countries (2). The Human Papillomavirus Virus (HPV) has been implicated in >95% of cervical cancers, therefore this disease is preventable through vaccination and screening. Both HPV vaccine and screening would avert 5.2 million cases, 3.78 million deaths, and 22.0 million disability-adjusted life years over the lifetime of the intervention cohorts over a 10-year program at the cost of US$2.2 million (3). Unfortunately, access to the vaccine is very sparse in low-income and lower-middle-income countries (LMIC). In August 2020, the 73^rd^ World Health Assembly passed a global resolution calling for the elimination of cervical cancer. This mandate comes 14 years after the introduction of HPV vaccines in the market. Currently, there are three vaccine types available: 9-valent HPV vaccine (Gardasil, 9vHPV), quadrivalent HPV vaccine (Gardasil, 4vHPV), and bivalent HPV vaccine (Cervarix, 2vHPV).

To eliminate cervical cancer as a public health problem there must be less than four cases per 100,000 women. A global strategy for elimination using vaccination and screening (primary prevention) and treatment of pre-cancers (secondary prevention) has been developed and approved by the WHO member states (4). As reported by the WHO Cervical Cancer Elimination Modeling Consortium (CCEMC), 90% of HPV vaccine coverage of girls (by 15 years of age) can be achieved through 70% coverage of screening followed by 90% uptake of treatment by 2030, leading to the elimination of cervical cancer globally by 2100 (5). The World Health Organization (WHO) recommends targeting vaccination to girls before their sexual debut (age 9-13), because HPV is sexually transmitted. As of 2019, 100 countries around the world have introduced the HPV vaccine into their national schedules. Despite this, 100 countries only cover 30% of the global target population (6). The barriers to the implementation of a national vaccination program have been previously reported on, however, these studies have mainly focused on screening, and not prevention of cervical cancer and/or the discussion of probable solutions. Given the critical role of HPV vaccination to prevent millions of cervical cancer related deaths, this review aims to understand the barriers to implementing HPV vaccination in developing countries as well as to give innovative solutions to each impediment as evidenced by demonstration projects, pilot studies, and evidence-based research. This aggregate of data can help inform future countries’ implementation programs to further the mission to vaccinate 90% of eligible girls globally by 2030. By investigating possible barriers to complete vaccination, fully informed solutions can be made to increase the efficacy of the campaign.

## Methods

To create the review, a six-step process was completed. The process included formulation of the review question, defining inclusion and exclusion criteria, developing a search strategy, selecting studies, extracting data, and analyzing and interpreting results.

### Search strategy

An electronic search was conducted using the Boolean terms “OR” and “AND”. Keywords used in the search are papillomavirus vaccines [Mesh], HPV, prevention, developing countries [Mesh], barrier, methods, and intervention. PubMed, Embase, and Cochrane Database of Systemic Reviews were systematically searched for the terms. The database search was supplemented by a hand search of Google Scholar using the same keyword search terms. Related articles were found through citation searching and added to the pool of articles to be screened.

### Inclusion and exclusion criteria

**Figure 1:**
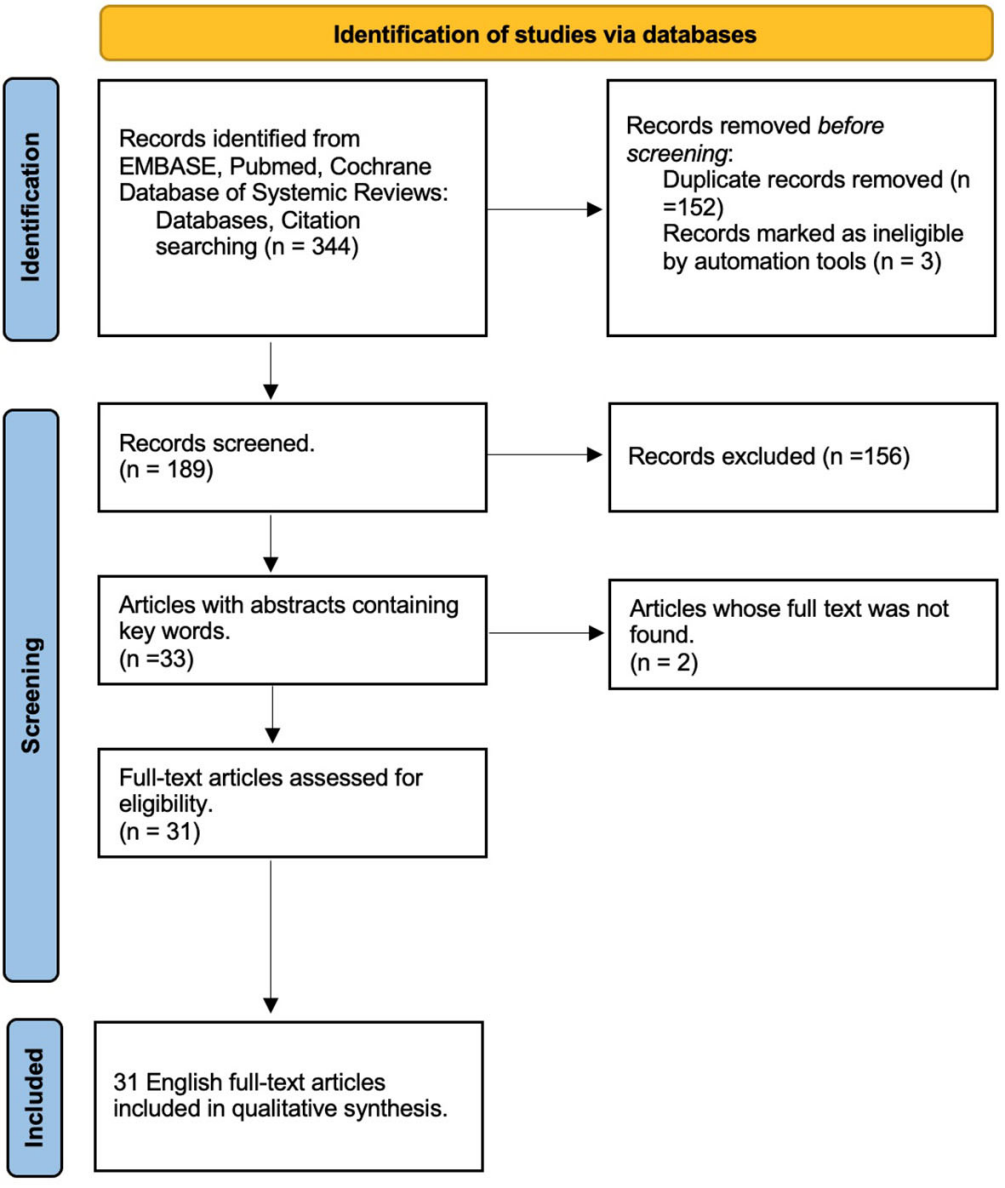
Study selection. *From:* Page MJ, McKenzie JE, Bossuyt PM, Boutron I, Hoffmann TC, Mulrow CD, et al. The PRISMA 2020 statement: an updated guideline for reporting systematic reviews. BMJ 2021;372:n71. doi: 10.1136/bmj.n7113/07/2024 00:47:00.

Based on the research questions, inclusion and exclusion criteria were created. Included were editorials, case studies, and full articles written in the English language that gave information on HPV vaccine programs and discussed the barriers and solutions involved in the creation of a national vaccine implementation program. The article must provide information on a developing or low- or middle-income country. Articles written before 2006 were excluded because the HPV Vaccine was not commercially available. Articles were included until March 1^st^, 2021. Articles that focused on evidence from high-income countries were excluded. Based on the abstract, each article was screened for relevant information. Country income groups were defined using the *World Bank Classification*, which categorizes countries according to their gross national income (GNI) per capita. Those that had full articles available were entered into the Endnote library. There were 189 articles screened and 31 articles included in this review (**Figure 1**).

### Data collection

An excel sheet was created to capture the following data from each article: author, year of publication, type of article, barriers to HPV vaccine implementation in the developing country, and solutions to the barrier. After careful reading of the article, data related to each category listed were entered into the excel sheet. Constant comparative analysis was conducted to identify thematic categories. Findings were reported according to The PRISMA 2020 statement: an updated guideline for reporting systematic reviews (7).

## Results

**Figure 2:**
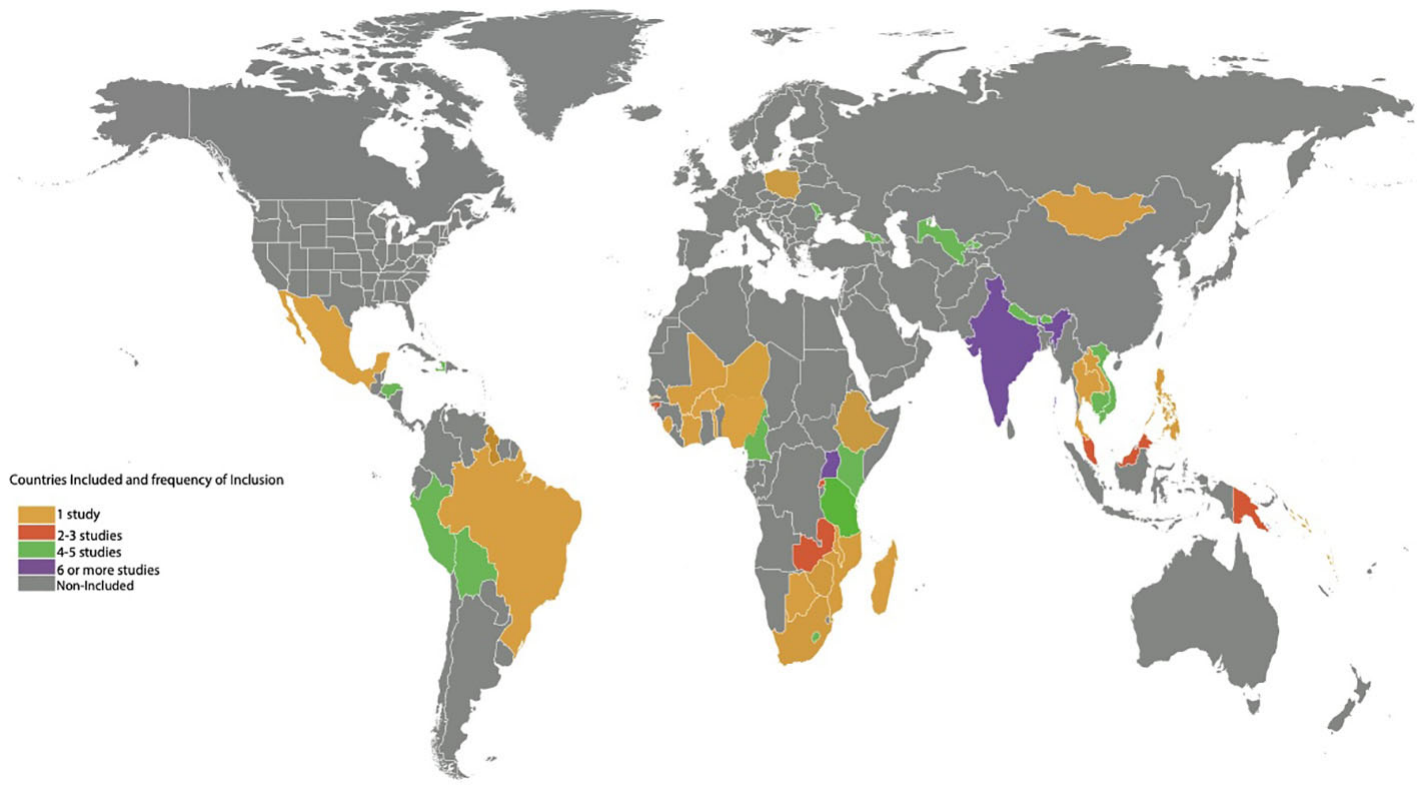
Heat map illustrating the distribution and location of countries Included.

A total of 31 research articles spanning 48 countries (n=48), geographic regions (Latin America/Caribbean), and 6 continents (Africa, Asia, Europe, Australia/Oceania, North America, and South America) were included in this review (**Figure 2**). Although 33 articles contained the key search terms, the statistical values presented were based on 31 studies. Two studies with missing full text manuscripts were excluded. The majority of these studies were systematic/mini reviews (25.8%), editorials (22.6%), and observational studies (16.1%) (**Tables 1**, **2**).

**Table 1:** Selected studies of HPV vaccination programs in developing countries and the associated barriers discussed.

Category	Author(s)	Year	Study design	Barrier(s)discussed
**Cost**	The Lancet	2008, 2011	Editorial	Pricing of HPV vaccines at the individual and government level limits uptake
	Adams P	2012	Editorial	
	Batson A, Meheus F, Brooke S	2006	Review	Lack of financial incentive to motivate rapid HPV vaccine development
	Ladner J, Besson MH, Hampshire R, Tapert L, Chirenje M, Saba J	2012	Observational study	Participating HPV programs are responsible for covering costs related to storage, administration, and community outreach
	Garnett GP, Kim JJ, French K, Goldie SJ	2006	Editorial	Patient/individual costs association with vaccination
**Mistrust in political and healthcare systems**	Agosti JM, Goldie SJ	2007	Editorial	Mistrust in government and healthcare initiatives results in perception of HPV vaccine as a tool to control fertility
	Ladner J, Besson MH, Audureau E, Rodrigues M, Saba J	2016	Observational study	Providing free vaccination leads to parents’ suspicion about hidden government agenda
**Low knowledge of HPV and its correlation to Cancer**	Bharadwaj M, Hussain S, Nasare V, Das BC	2009	Commentary	Parents of school-aged girls unaware of HPV. Belief that children are not at increased risk for HPV related cancers
	Biellik R, Levin C, Mugisha E, LaMontagne DS, Bingham A, Kaipilyawar S	2009	Review	Baseline survey study in Kenya demonstrates poor parental knowledge about HPV vaccine and associated cancer prevention benefits
	LaMontagne DS, Barge S, Le NT, Mugisha E, Penny ME, Gandhi S,	2011	Editorial	Low knowledge of parents about HPV, its relation to STI and cancer
	Natunen K, Lehtinen J, Namujju P, Sellors J, Lehtinen M	2011	Editorial	Lack of knowledge by parents and girls about HPV, its associated cancer risk, and the benefits of vaccination.
**Social stigma (“STI” vaccine)**	Ladner J, Besson MH, Audureau E, Rodrigues M, Saba J	2016	Observational study	Parents and adolescent stigma about the societal implication receiving an “STI” related vaccine
	Wong LP	2009	Observational study	Community concern about “Halal-ness” of STI-associated vaccine and its health impact
**Inadequate training/knowledge of medical professionals**	Gallagher KE, Howard N, Kabakama S, Mounier-Jack S, Burchett HED, LaMontagne DS,	2017	Descriptive synthesis	Poor investment by governments in HPV vaccine programs results in inadequate training of healthcare professionals to ensure accurate information sharing with parents/girls.
	Chidyaonga-Maseko F, Chirwa ML, Muula AS	2015	Systematic review	Health systems lack sufficient education of health professionals and the vaccine team
**Poor vaccine implementation strategy (target age group, school-based delivery system, reaching-of-school girls)**	Gallagher KE, Howard N, Kabakama S, Mounier-Jack S, Burchett HED, LaMontagne DS,	2017	Descriptive synthesis	School absenteeism creates an obstacle for school-based implementation HPV vaccine programs
	Jennings MC, Loharikar A	2018	Commentary	School based delivery system is inequitable for out-of-school girls in more remote communities
	Kane MA, Serrano B, de Sanjosé S, Wittet S	2012	Commentary	Poor selection of the appropriate vaccine target group has a negative impact on implementation strategies.
**Limited governmental involvement/lack of technical capacity**	Pollack AE, Balkin MS, Denny L	2006	Review	Invisible diseases like cervical cancer (delayed effect) do not generate significant political will to drive resource allocation for prevention programs to prevent disease in subsequent generations.
	Jit M, Demarteau N, Elbasha E, Ginsberg G, Kim J, Praditsitthikorn N	2013	Consensus review	LMICs lack the technical capacity and strategic economic data analysis to design sustainable HPV vaccine programs and rollouts.
	Howard N, Gallagher KE, Mounier-Jack S, Burchett HED, Kabakama S, LaMontagne DS	2017	Systematic review	Inadequate political commitment and investment in new and existing HPV vaccination programs
**Parental/caregiver concerns about vaccine-related side effects/misinformation**	Chidyaonga-Maseko F, Chirwa ML, Muula AS	2015	Systematic review	Parental fear about fertility and sexual sequelae of HPV vaccine
	Ladner J, Besson MH, Audureau E, Rodrigues M, Saba J	2016	Observational study	
**Multi-dosage vaccination schedule/poor adherence**	Baussano I, Lazzarato F, Ronco G, Dillner J, Franceschi S	2013		Multi-dosage vaccination schedule is a barrier to HPV vaccine uptake and adherence of disadvantaged communities
	Pollack AE, Balkin MS, Denny L	2006	Review	Patient compliance is impacted by the need to return for multiple doses of HPV vaccines, especially in a resource limited setting.
	LaMontagne DS, Barge S, Le NT, Mugisha E, Penny ME, Gandhi S,	2011	Editorial	
**Poor population wide communication**	Gallagher	2017	Descriptive Synthesis	Poor and negative public messaging about a sexually related vaccine results in parental concerns that vaccine may promote sex.
	Wong LP	2009	Observational study	Insufficient dissemination of positive messaging and information about HPV vaccine

**Table 2:** Major barriers to successful vaccine implementation and proposed solutions.

Barriers	Solutions
**Low knowledge of HPV and its relation to cervical cancer**	Community sensitization (30, 39, 40)
**Low Medical professional knowledge**	Timely education of medical professionals (15, 39)
**Cost**	Price negotiation, GAVI alliance (23, 24, 26, 30)
**Completion of vaccine series**	Make a part of existing immunization program (8, 15)
**Vaccine target age and group**	Target by grade rather than age (30)
**Cold storage**	Controlled temperature chain (36)
**Mistrust in government healthcare initiatives**	Community sensitization and advocacy (31, 35)
**Reaching out-of-school girls**	Employ peer tracking (17)
**Lack of political will**	coordination of advocacy groups from diverse backgrounds (23, 35)
**Parental concerns of side effects**	Employ informed consent with an “opt in” protocol (28)
**Poor communication**	Employ well trained media professionals (11, 14, 31)
**“STI” vaccine**	Emphasize it as a cancer vaccine (11, 14)

For studies highlighting HPV vaccine challenges in specific countries (n=16), majority focused on nations in Africa (n=7) followed by Asia (n=3) and South America (n=2). There were 4 studies reporting challenges in multiple countries (n=4). The average human development index (HDI) of the represented countries was 0.64. Most of these studies focused on vaccine initiatives for young girls and adolescents with ages ranging from 9 to 18 years (35.5%).

### Education/Literacy

A lack of knowledge and low literacy about the benefits of the vaccine is a major reason for low vaccine uptake in developing countries. For example, one study of Indian parents of 9 to 16-year-old schoolgirls suggested low literacy among parents, with most of them being unaware of HPV and harboring the belief that their children are not at risk of acquiring HPV “as they come from a good family.” They also have misconceptions about the vaccine, with parents suspecting that the vaccine itself may cause HPV infection in their children (8). In 2016, a study to survey awareness and attitude towards HPV and the HPV vaccines among market women in Bodija Market in Nigeria revealed that all survey respondents were sexually active with a majority having multiple sexual partners. Awareness of the HPV vaccines was 1.2% among participants, with 92.4% having a positive attitude toward the vaccine and 91.8% willing to take the HPV vaccine (9) after learning about its benefits. In Kenya and Malaysia, baseline knowledge of HPV infection risk was low overall. However, after learning about the vaccine, 95% of mothers were willing to allow their daughters to be vaccinated (10). Another study suggested that parents are more receptive to HPV vaccine if it is portrayed as a “cancer vaccine” (11). Parental concerns include possible side effects such as infertility, early sexual onset, increased sexual activity, and vaccine safety (12). To improve health literacy and acceptance among parents, the vaccine needs to be described in a manner that is acceptable to parents. Emphasis needs to be placed on the vaccines ability to prevent a cancer that is caused by a virus. Even though HPV is a sexually transmitted infection- parents are more likely to accept vaccination for their children if it is presented as a vaccination for prevention of cervical cancer. In addition, creating adequate community sensitization is important to avoid/decrease misinformation about the vaccine, therefore the timing and introduction of the vaccine is critical to improve vaccine uptake.

### Social and cultural acceptability

A successful vaccine program will need to address the complex interplay between familial developmental and psychosocial factors that influence adherence. Adolescents are a special population with unique barriers. The barriers include a lack of self-efficacy, stigma, and an inability to consider future medical consequences (13). Furthermore, narrowly targeting adolescent females could create additional barriers. While targeting young girls is a cost-effective strategy, concerns arise regarding fears that girls can become infertile due to the vaccine or that it may increase sexual promiscuity. Vaccine acceptance therefore depends on both the caregivers’ and adolescents’ perceived vulnerability of getting the disease and their willingness to be vaccinated (14). To mitigate this issue, in the United States, the vaccine is approved for adolescents regardless of gender. However, uptake in LMICs has been challenging for a variety of reasons.

Some challenges include determining the appropriate age of children to be vaccinated and inadequate training of vaccine administrators. Efforts targeting school age children have been met with incomplete registration of schools by the ministry of education at the district level and high rates of absenteeism (15). For example, in India, Peru, Uganda, and Tanzania, even though school attendance is reported to be high in all countries, school absenteeism is identified as the main reason for deficiencies in vaccine programs (15, 16).

One program developed an innovative solution to provide second and third vaccine doses in small communities, including communities with limited cell phones. They implemented a method of peer tracking of girls by soliciting the help of girls who were getting vaccinated, to locate other girls in that community who might need additional doses (17). Other vaccine program developers report comprehensive social mobilization of the whole community as successful initiatives including face-to-face meetings with local credible influencers (health workers, teachers, religious leaders, community elders) (15). For example, the use of school programs in combination with existing child public health days in Uganda for the purpose of reaching out to schoolgirls achieved 52.6% coverage (11). Rwanda used community involvement to identify girls who were absent from or not enrolled in school combined with a national sensitization campaign prior to delivery of the first dose and achieved over 93% coverage for all three rounds of vaccination (18). As a result, Rwanda is on track to meet the United Nations goal to reduce premature mortality from non-communicable diseases by one-third by 2020 (19). Another innovative strategy that has some reported benefit is a catch-up program. In Guinea, where the HPV prevalence is 58%, a catch-up vaccination program of eight cohorts of women was estimated to reduce HPV 16/18 prevalence by 50% in women less than 35 years of age by as much as 5 years compared to targeting 11-year-old girls only (20).

### Communication

Effective community mobilization activities require implementation at least one month prior to vaccination and should utilize multiple channels. These channels include celebrity champions, WHO and government endorsement, sponsorship from community leaders, and community engagement. The most effective messages emphasized cancer prevention, national and global endorsement, and vaccine safety. In addition, face-to-face communication with credible influencers (teachers, health-workers, community leaders) and clear consent procedures consistent with those used for routine immunization were found to be more successful (21). Many countries found that it is good practice to have well trained media spokespersons involved early in the planning process before introduction of vaccine initiatives (22). Three program managers reported that asking for written consent and providing vaccines free of charge was viewed negatively by patients and heightened their suspicion about HPV vaccines (17); therefore, information strategies designed to address misperceptions are critically important (23). In rural areas lack of information was more pervasive, whereas in urban areas misinformation was more common, therefore, program managers emphasize the importance of providing effective messaging, sensitization strategies and coupling HPV vaccination with other health interventions” (17).

### Cost

In developing countries, cost is a major barrier to vaccine roll-out. HPV vaccine is the most expensive childhood immunization in the world with an estimated cost of US$360 for the three required doses of Merck’s Gardasil and US$335 for a complete course of GlaxoSmithKline’s Cervarix (24). To improve access and make vaccines more affordable, key stakeholders have to be engaged. Ministries of Education and international NGOs play vital roles in supporting human resources to facilitate successful immunization programs in these regions. For example, eighteen programs received financial support from and had significant involvement of international NGOs (17) for successful implementation. Additionally, the Global Alliance for Vaccines and Immunization (GAVI), a global health partnership of governments from industrialized and developing countries, UNICEF, WHO, the World Bank, and the Bill and Melinda Gates Foundation (25, 26) was launched in 2000 to improve the access of developing countries to immunization and vaccines.

Rwanda became the first country in Sub-Saharan Africa to create a national prevention program for cervical cancer, which included both HPV vaccination for girls aged 12-15 years and HPV testing for women between 35 and 45 years. This was possible due to a three-year donation of 2 million doses of Gardasil by Merck and the agreement to provide Rwanda with discounted access price to the vaccine after 3 years (27). A comprehensive study set at different institutions around the world used hypothetical models in low and middle-income countries and found that the rate of discounting, vaccine price, and HPV prevalence influenced cost-effectiveness (28). Furthermore, price negotiation is a successful way to reduce prices for countries that cannot afford them. For example, the Pan American Health Organization secured a price of $16.95 per dose for members of its Expanded Program for Immunization Revolving Fund (27). A study of options for cervical cancer control in Brazil found that at a cost of $5 per dose, the cost-effectiveness ratio associated with adolescent vaccination would be less than $150 per year of life saved and vaccination combined with screening women at least three times during their lifetime would create a very cost-effective intervention. For countries with a gross domestic product of less than $1,000 per capita, the per-dose cost needs to be as low as $1 to $2 to make vaccines affordable (29). Cost sharing is another method to decrease the cost of vaccine roll-out programs. Combining HPV vaccination with other age-related services will reduce the cost and burden on healthcare systems of delivering separate interventions. Delivering the vaccine alongside other packages such as vitamin A and deworming medications distributed by health workers during the twice-yearly Child Days Plus program decreases cost of forming a new program. Delivering it at the same time each year helps with planning of health worker’s time and space, organizes the cold chain, and reduces disruption in schools (30).

Another potential option to improve vaccine access in LMICs is with advanced market commitments (AMCs) – an innovative pull mechanism. AMCs are financial commitments to subsidize the future purchase of a vaccine that is not yet available if the vaccine meets certain standards and is demanded by developing countries. It helps stimulate manufacturers to make additional investments in the development and production of the desired vaccines and accelerates their introduction in developing countries (25). The “Pull” mechanisms provide greater confidence in future sales and their ability to generate a return on investment. An example of a pull mechanism is GAVI’s multi-year commitment to purchase underused vaccines for the poorest countries (25). Other innovative solutions include the “Push” mechanism which is a direct investment in basic research, product development, or production capacity. For some LMICs, a partnership between the public and private sectors allows entities to share the risks and costs of developing, scaling up, and introducing priority vaccines.

### Vaccine delivery

HPV vaccine delivery requires creating a new health routine for a new target population and is a major challenge. Understanding the population of eligible girls before vaccination though challenging and costly is necessary because existing population data can be unreliable or inaccurate. Implementing a two-dose vaccination schedule was easier and less costly than a three-dose schedule. When given all the doses within one school year, dropout was minimized and there was increased coverage. In addition, providing a second vaccination opportunity successfully reached girls and parents who initially refused or were absent (31). In 2009, The Ugandan government piloted an HPV Vaccine rollout program and found that they reached more girls by selecting them by grade rather than age (30). Vietnam tested two delivery strategies, one through school and another through community health centers, and achieved 98.00% coverage for that specific community with both methods (11). Therefore, mixed models that incorporate both schools and health facilities have better coverage than models using only one method (32). Other methods to reduce barriers to access include offering vaccinations regionally (33) using a single dose mechanism, A single-dose schedule, if effective, will offer gains in cost and simplicity of delivery. Some studies show that the single dose may be potentially protective against HPV infection for healthy young girls, but the existing evidence is not robust enough for global recommendation (34, 35).

### Infrastructure

Many LMICs lack the infrastructure to store acquired vaccines. Cold storage presents a unique barrier, but one way to circumvent cold storage is using a process called a controlled temperature chain (CTC). A CTC promotes suitable vaccines at temperatures outside of the traditional cold chain of 2-8 degrees Celsius. Gardasil has already been licensed and prequalified for CTC. This method saves costs associated with developing the infrastructure for cold storage. CTC vaccines also enables easier outreach to schools and rural communities by extending supply chains, thereby improving immunization coverage and equity (36).

## Discussion

In 2011, a summary of HPV Vaccination in 45 LMICs discovered that 1.7 million girls were reached and 1.4 million were fully vaccinated (31), highlighting that these afore mentioned efforts to vaccine delivery can be effective. However, the numbers of children needing vaccinations are still low. HPV presents a unique challenge because of the delayed impact of cervical cancer (11); therefore, achieving the goal of eliminating cervical cancer requires coordination among diverse stakeholders who address sexual and reproductive health, adolescent health, immunization, and cancer, without competing for resources. Creative solutions to alleviate constraints in low-resource settings include implementing an HPV faster protocol, using one visit to vaccinate women aged 9-45 irrespective of HPV infection status and screen women above 25-30 using a validated HPV test as part of their initial visit (37). Other priorities include financial investment and resource allocation for tailored community education programs and to health works, as well as decentralized policy adaptations to meet the needs of rural populations. Additionally, educational interventions and support of these interventions are critical to guarantee the success of national HPV vaccine implementation programs (38, 39). Rwanda serves as the best-case example as it became the first low-income country in the world to introduce HPV Vaccine into its national program in 2011. With strong leadership from its First Lady and partnership with public and private sectors, they employed evidence-based mobilization efforts and reported between 93% to 96% full course coverage (22). Governments may have competing health priorities and must weigh the benefits and costs of the HPV vaccine with other interventions. The rotavirus vaccine, for example, has been shown to save a similar number of lives over the population’s lifetime (36).

This systematic review has several strengths, including the utilization of a comprehensive search strategy, inclusion of research articles spanning multiple countries and continents, and adherence to a standardized and rigorous methodology process for study identification, data extraction, and synthesis. Furthermore, the review provides an extensive examination of barriers to HPV administration and offers sustainable and innovative solutions to address these challenges. However, inherent limitations exist within the systematic review process. First, there is potential for publication bias, with inclusion of only articles written in English. This could result in exclusion of relevant non-English studies, and therefore limit the study findings. Additionally, the variability in study quality, methodology, and design could introduce inconsistency in the extraction and synthesis of evidence. Lastly, tools to categorize study quality can bias towards selecting studies with positive associations with vaccine programs. Despite these limitations, the findings of this article offer valuable insights and practical strategies for policy makers and public health officials in LMICs. The evidence-based approach of this paper informs future implementation programs, with the goal of improving vaccine coverage and cervical cancer elimination in developing countries.

## Conclusion

Our review suggests that implementation of HPV Vaccination programs may be feasible in low-resource settings provided that the health system structure for immunization and national and international financing options are well understood. Further research can analyze the cost and benefits of vaccinating both girls and boys in low-income countries. Low-income countries continue to create innovative solutions to increase HPV vaccine uptake with the ultimate goal of decreasing the morbidity and mortality from cervical cancer.
